# The consistency and efficacy of optical coherence tomography for the evaluation of ocular torsion angle in children

**DOI:** 10.3389/fped.2025.1519017

**Published:** 2025-01-30

**Authors:** Rongjun Liu, Jingjing Zhao, Kaili Yu, Dehai Zhu

**Affiliations:** Department of Pediatric Ophthalmology, Peking University First Hospital, Peking University Pediatric Visions Research Center, Beijing, China

**Keywords:** ocular torsion angle, OCT, fundus, children, strabismus

## Abstract

**Purpose:**

To explore the consistency and efficacy of optical coherence tomography (OCT) for the evaluation of ocular torsion angle compared to fundus photography in children.

**Method:**

Patients who had undergone fundus photography and OCT within 1 day were included in this study. The fundus photographs were taken using a fundus camera and then imported into the Adobe Photoshop 2021 software. The fovea-disc angle (FDA) was calculated manually. OCT was used to automatically detect the centers of the optic disc and fovea, and the software then calculated the FDA on the same device. The means and ranges of variables were calculated in this study. Group differences were assessed using the paired *t*-test. Statistical significance was determined when *P*-values were <0.05. Bland–Altman plots were constructed to verify the agreement of the FDAs measured by fundus photography and OCT respectively.

**Results:**

A total of 100 patients were included and the objective measurements of the ocular torsion angles via OCT and fundus photography were similar. The mean FDA of 32 patients aged 2–6 years old was −7.84 ± 4.67° via fundus photography and −6.71 ± 6.19° via OCT. The mean FDA of 68 patients aged 6–18 years old was −8.47 ± 5.22° via fundus photography and −8.97 ± 5.41° via OCT. According to the receiver operational characteristic (ROC) curves of the FDA for diagnosing ocular extorsion, the area under the ROC curve was greatest for OCT (0.943, 95% CI: 0.902–0.984), followed by fundus photography (0.92, 95% CI: 0.86–0.979). With the Youden method, the optimal cut-off point for diagnosing ocular extorsion with OCT was −6.35°. OCT demonstrated a sensitivity of 100% and a specificity of 49.2%. Furthermore, the optimal cut-off point for diagnosing ocular extorsion with fundus photography was −6.5°.

**Conclusion:**

The comparison of FDAs showed good agreement between fundus photography and OCT. Thus, OCT can evaluate ocular torsion angle effectively in children.

## Introduction

Ocular torsion, also known as cyclodeviation, is a condition characterized by the eye rotating abnormally around the visual axis. It is caused by an imbalance of strength between the ocular muscles that are responsible for intorsion such as the superior oblique and superior rectus and those responsible for extorsion such as the inferior oblique and inferior rectus (1). It is common in patients with primary or secondary inferior oblique muscle overaction (IOOA), superior oblique muscle palsy, intermittent exotropia (IXT), and skew deviation (2). This misalignment can be either extorsion or intorsion of one or both eyes in children and it significantly affects their binocular vision development (3). Clinical assessment of subjective and objective anatomic torsion is an important adjunct for the diagnosis and treatment of cyclovertical strabismus (4).

Various diagnostic techniques are available to measure ocular torsion, including subjective tests such as the Maddox rod test, the Bagolini lens test, and the Lancaster red-green test. Furthermore, fundus photography is commonly used (5). The ocular torsion angle is determined by the angular displacement below a horizontal line connecting the center of the optic disc and the center of the fovea. In recent years, research has focused on understanding the etiology and pathophysiology of ocular torsion and developing more precise diagnostic approaches. Heidelberg Engineering (Heidelberg, Germany) developed the SPECTRALIS optical coherence tomography (OCT) platform, equipped with the Glaucoma Module Premium Edition (GMPE) software and an anatomic positioning system, that is able to record position information of the retina and optic disc by detecting the centers of the fovea and the optic disc automatically. This in turn allows for automated measurement of the ocular torsion angle (6). Several researchers have reported the application of OCT to evaluate ocular torsion angle (7, 8). However, ocular torsion is detected at a significantly higher rate in children than in adults and is underdiagnosed in clinical examinations as the children are often asymptomatic (9). Therefore, in this study, we aimed to explore the consistency and efficacy of OCT in the evaluation of ocular torsion angle in children compared to fundus photography.

## Methods

### Study design and eligibility

A retrospective analysis was performed at the Department of Pediatric Ophthalmology in Peking University First Hospital from June 2022 to December 2023. The study was performed in accordance with the Declaration of Helsinki and with approval from the ethics committees of Peking University First Hospital.

We included children aged 2–18 years old who had undergone fundus photography and OCT within 1 day and excluded those with any other ocular diseases and ocular surgery histories. We extracted data from their medical records including age, sex, ocular muscle movement examinations, and ocular torsion type. Images of the left eye of each child were used in the study. A prism cover test was performed to record the horizontal and vertical deviation in prism diopters. Subgroup analyses were performed between patients aged from 2 to 6 years old and those aged 6 to 18 years old, between patients with ocular extorsion and non-ocular torsion, and between patients with IOOA and without IOOA.

### Measurements of the ocular torsion angle

**Figure 1:**
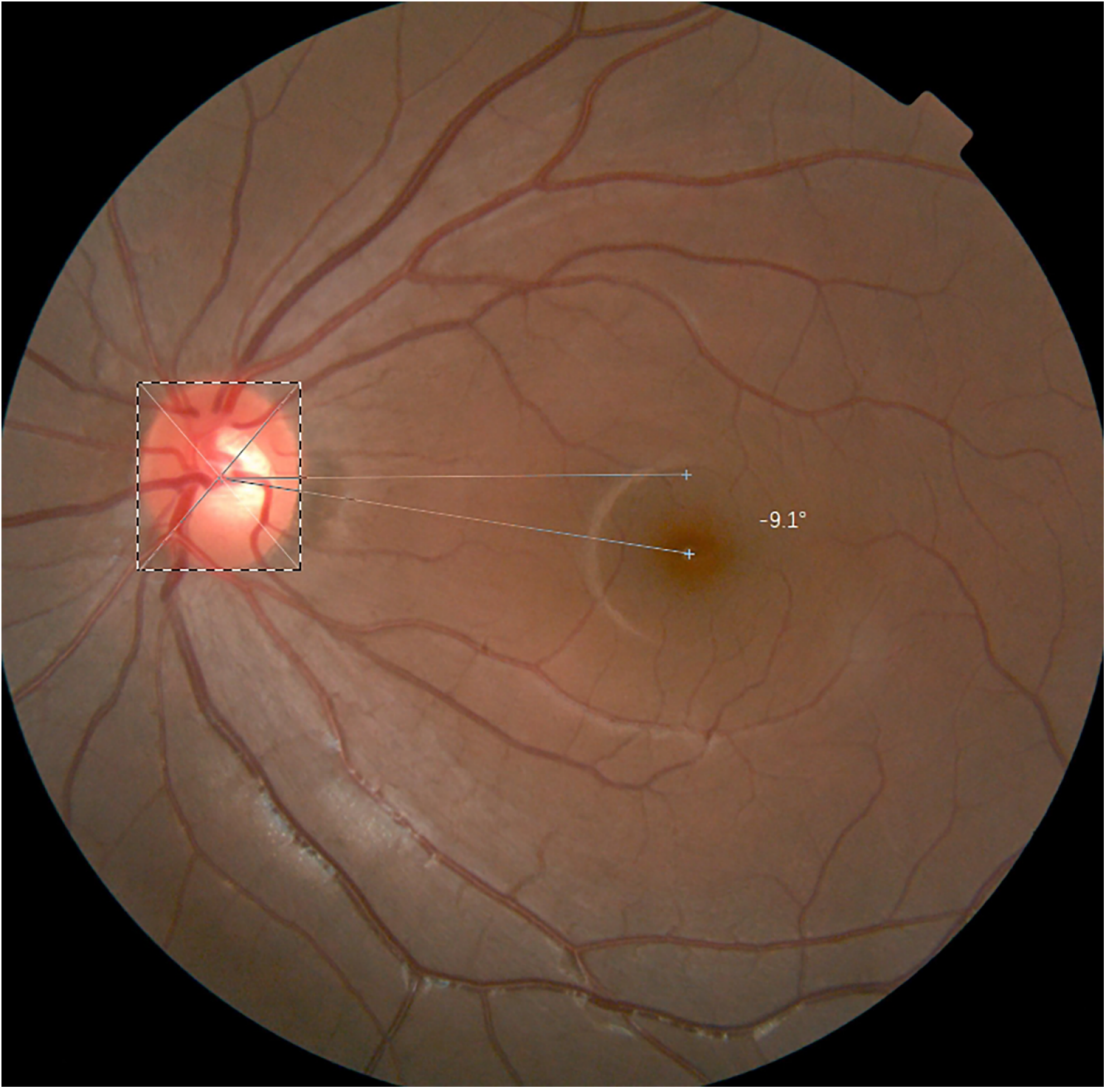
The fundus photography was manually located the anatomical center of optic disc and macula fovea in Adobe Photoshop 2021 software and the FDA in fundus photography is −9.1°.

The method for measurement using fundus photography was followed as described in the literature. We took the fundus photographs using a non-mydriatic fundus camera. In clinical settings, qualitative assessment of ocular torsion is widely conducted using fundus photography. Non-ocular torsion is defined as the macula fovea being located in the lower part of the optic disc. Ocular extorsion is defined as the macula fovea being located lower than the bottom edge of the optic disc (as shown in the Supplementary Material). All the results relied on the observation of the clinician. The quantitative results were calculated using Adobe Photoshop 2021 software. The fundus photographs were imported into the Adobe Photoshop 2021 software, which was used to manually locate the anatomical center of the optic disc and macula fovea. The ocular torsion angle was calculated by the fovea-disc angle (FDA), which is formed by the connection between the macula fovea and the center of the optic disc and the horizontal line through the center of the optic disc (as shown in Figure 1). The software measured the degree of the FDA automatically. It took approximately 5 min to complete a measurement of the ocular torsion angle using fundus photography.

**Figure 2:**
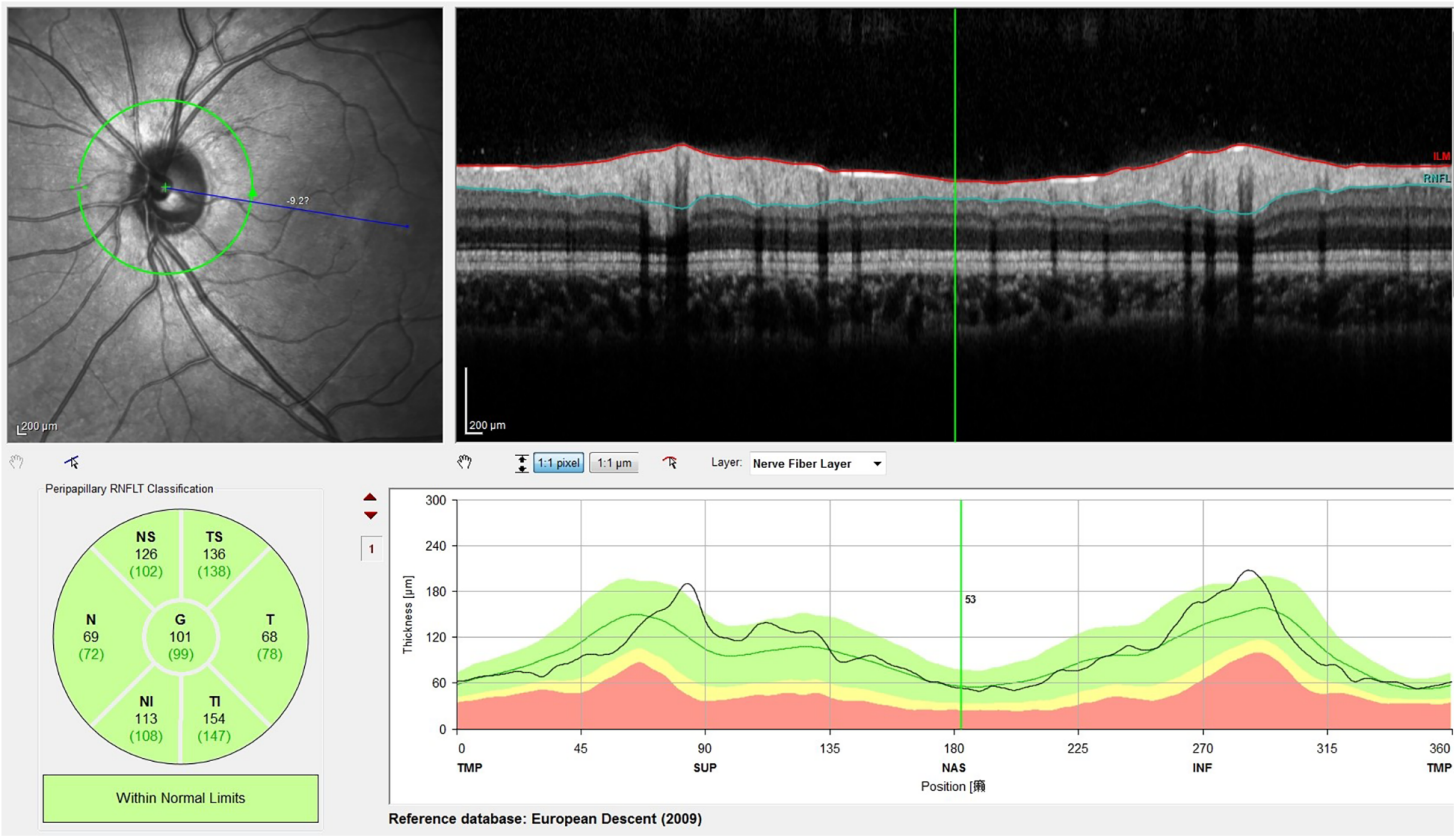
OCT scans the retina, and GMPE automatically detects the centers of the optic disc and macula fovea, and then the software calculates the FDA. The picture shows an FDA of −9.2°.

For OCT, we used the SPECTRALIS GMPE-based OCT. Briefly, OCT scans the retina, the GMPE system automatically detects the centers of the optic disc and fovea and the software then calculates the FDA on the same device (as shown in Figure 2). It took approximately 3 min on average to complete a measurement of the ocular torsion angle using OCT.

### Statistical analyses

The means and ranges of variables were calculated in this study. Group differences were assessed using the paired *t*-test and independent *t*-test. A Bland–Altman plot was used to compare the differences in the ocular torsion angle measurements between OCT and fundus photography. For all analyses, *P-*values and two-sided 95% confidence intervals for point estimates were reported. Statistical significance was determined when *P*-values were <0.05.

## Results

### Participants’ data

A total of 100 patients were included in this study. All patients were enrolled with one eye, including 47 boys and 53 girls aged between 2 and 18 years old with an average age of 9.15 ± 4.11 years old. There are 80 patients with strabismus including horizontal and vertical strabismus and 20 patients without strabismus.

### Comparisons of ocular torsion angles calculated by different measurement methods

The objective measurements of ocular torsion angles via OCT and fundus photography were similar. The mean FDA of 32 patients aged 2–6 years old was −7.84 ± 4.67° via fundus photography and −6.71 ± 6.19° via OCT. The mean FDA of 68 patients aged 6–18 years old was −8.47 ± 5.22° via fundus photography and −8.97 ± 5.41° via OCT. The results are shown in Table 1. The mean FDA of 39 extorsion eyes was −12.64 ± 3.65° via fundus photography and −13.49 ± 4.08° via OCT. The mean FDA of 61 non-ocular torsion eyes was −6.30 ± 2.87° via fundus photography and −5.55 ± 3.78° via OCT. The results are shown in Table 2. Bland–Altman plots were constructed to verify the agreement of FDAs measured by fundus photography and OCT. The comparisons showed good agreement between fundus photography and OCT.

**Table 1:** Comparisons of the ocular torsion angle via fundus photography and OCT in different ages.

	Total	2–6 years old	6–18 years old
Total (eyes)	100	32	68
FCP (°)	−8.28 ± 5.05	−7.84 ± 4.67	−8.47 ± 5.22
OCT (°)	−8.278 ± 5.73	−6.71 ± 6.19	−8.97 ± 5.41
*T*-value	−0.003	−1.76	1.370
*P*-value	0.998	0.088	0.175

FCP, fundus color photography; OCT, optical coherence tomography.

**Table 2:** Comparisons of the ocular torsion angle via fundus photography and OCT in extorsion and non-ocular torsion.

	Extorsion	Non-ocular torsion
Total (eyes)	39	61
FCP (°)	−12.64 ± 3.65	−6.30 ± 2.87
OCT (°)	−13.49 ± 4.08	−5.55 ± 3.78
*T*-value	1.436	−1.973
*P*-value	0.159	0.053

FCP, fundus color photography; OCT, optical coherence tomography.

### Comparisons of the ocular torsion angle in different grades of inferior oblique muscle overaction

The patients with IOOA were all found to have ocular extorsion. The grade of IOOA is distinguished by the distance between the lower limbus of the cornea and the lower palpebral margin of the eye with an internal upward rotation of 45° according to a study by Hunter and Parks (10). Thus, when the distance is over 1 mm, it is judged as IOOA 1+, 2 mm as IOOA 2+, 3 mm as IOOA 3+, and 4 mm as IOOA 4+. In this study, there are 71 patients without IOOA and their mean FDA was −7.94 ± 4.82° by fundus photography and −7.22 ± 5.15° by OCT. There are 29 patients with IOOA including 18 with IOOA 1+, seven patients with IOOA 2+, and four patients with IOOA 3+. The mean FDA was −10.01 ± 4.97° in the patients with IOOA by fundus photography and −11.72 ± 6.12° by OCT (as shown in Table 3).

**Table 3:** Comparisons of the ocular torsion angle in different grades of inferior oblique muscle overaction.

	Without IOOA	With IOOA	*F*-value	*P*-value
Total (eyes)	71	29		
FCP (°)	−7.94 ± 4.82	−10.01 ± 4.97	3.572	0.062
OCT (°)	−7.22 ± 5.15	−11.72 ± 6.12	14.52	<0.001

FCP, fundus color photography; OCT, optical coherence tomography.

### Diagnostic efficacy of FDA for ocular extorsion by different measurement methods

According to the receiver operational characteristics (ROC) curves of the FDA for diagnosing ocular extorsion, the area under the receiver operational characteristics curve (AUC) was higher for OCT (0.943, 95% CI: 0.902–0.984) compared to fundus photography (0.92, 95% CI: 0.86–0.979). Using the Youden method, the optimal cut-off point for diagnosing ocular extorsion with OCT was −6.35°. OCT demonstrated a sensitivity of 100% and a specificity of 49.2%. Furthermore, the optimal cut-off point for diagnosing ocular extorsion with fundus photography was −6.5°. Fundus photography demonstrated a sensitivity of 97.4% and a specificity of 52.5% (as shown in Table 4).

**Table 4:** Diagnostic efficacy of FDA via fundus photography and OCT for ocular extorsion.

	Cut-off values	Se (%)	Sp (%)	YI	AUC	AUC 95% CI lower	AUC 95% CI upper
FCP (°)	−6.5	97.4	52.5	0.499	0.92	0.86	0.979
OCT (°)	−6.35	100	49.2	0.492	0.943	0.902	0.984

AUC, area under the receiver operational characteristics curve; CI, confidence interval; Sp, specificity; Se, sensitivity; YI, Youden index.

## Discussion

Strabismus can be horizontal, vertical, cyclodeviation, or a combination of all three. Cyclodeviation, also called ocular torsion caused by an imbalance of strength between the ocular muscles that are responsible for intorsion and extorsion. It was first reported as part of the strabismus pattern when measuring overaction of the obliques (11). Most ocular torsion is caused by congenital factors, such as congenital superior oblique muscle palsy, but there are also some acquired factors such as vitreoretinal diseases, post-cataract surgery, or muscle suturing deviations during strabismus surgery (12).

In clinical settings, it is challenging to measure the ocular torsion angle using conventional methods, and the choice of surgical methods often relies upon experience. Therefore, it is important to explore new methods to quantitatively assess ocular torsion angle that would provide a precise standard for diagnosis and strabismus surgery design. Postoperative ocular torsion angle could also be used to evaluate the effect of a surgery method.

Clinical assessment of ocular torsion is typically obtained subjectively and objectively. Subjective measurements require a patient's interpretation of visual input which may not correspond with the anatomical position of the eye (13). Objective assessments are vital to reach an accurate diagnosis and make a surgical decision regarding the treatment of cyclovertical strabismus. Objective ocular torsion measurement is a routine procedure in strabismus examinations (14). However, the lack of clinical manifestations and subjective complaints have led to the underdiagnosis of objective ocular torsion. Clinical assessment of subjective or objective anatomic torsion is difficult at all ages but is especially so in children.

Several methods have been used to objectively measure ocular torsion, including artificial markers such as physical markers, corneal tattoos, scleral markings, a search coil, a tracking system on an excimer laser, iris pattern imaging, and fundus photography (15). Among these, fundus photography is the most common method used to evaluate ocular torsion. A study by Piedrahita-Alonso et al. (14) assessed the agreement between FDA and rotation of the retinal vascular arcades in measuring ocular torsion. The results showed that rotation of the retinal vascular arcades was not a substitute for FDA which is the gold standard for ocular torsion evaluation. Objective measurements are vital to reach an accurate diagnosis and make a surgical decision regarding the treatment of cyclovertical strabismus. According to the fundus image, if the ocular intorsion is diagnosed with the macula fovea reflection spot is above the straight line through the center of the optic disc. Ocular extorsion is diagnosed if the macula fovea reflection spot is lower than the bottom of the optic disc (16). However, fundus photography does not provide a quantitative assessment of the ocular torsion angle directly and is difficult in patients with small pupils or optic media opacities. A study by Wang et al. (17) developed deep learning models using synthetic fundus images to assess ocular torsion and ocular torsion angle, however, the application needs further research. For this reason, quantitative assessment of ocular torsion angle is not widely used to assist in clinical diagnosis and treatment.

As technology has developed, OCT has become a routine examination in clinical settings and the application of OCT still needs to be intensively studied. GMPE-based OCT with an anatomic positioning system is able to record positional information for the retina and optic disc by detecting the centers of the fovea and the optic disc. It could be useful to automatically calculate cyclotorsion and compare it with conventional fundus photographs (7). A previous study showed that the objective measurements of the ocular torsion angle via OCT and fundus photography were similar (18). As expected, subjective torsion measured using a synoptophore was smaller than the ocular torsion angle when measured using both fundus photography and OCT (8). However, data on the efficacy of OCT in the evaluation of the ocular torsion angle is still lacking. In our study, we calculated the mean ocular torsion angles of extorsion patients and normal people, −13.49 ± 4.08° and −5.55 ± 3.78°, respectively. The cut-off value when using OCT in the diagnosis of ocular extorsion was −6.35°. These results are consistent with a previous study by Kang et al. (7) that showed that the ocular torsion angle evaluated by OCT was −6.16 ± 3.50° in patients with intermittent exotropia, −6.91 ± 1.12° in patients with superior oblique palsy, and −5.72 ± 3.20° in normal controls. A study by Lee et al. (19) was the same as our study as it suggested that as the degree of IOOA increases, the ocular torsion angle becomes larger.

Ocular torsion is common in children with horizontal strabismus or overaction of the inferior obliques, even if in a V pattern (20). The high incidence of torsion in children should be taken into consideration when planning strabismus surgery. However, few children had subjective complaints of torsion and traditional methods such as Double Maddox Rods or Lancaster red-green testing depend on their cooperation (9, 21). For objective assessment, a study investigated ocular torsion and its variation using fundus photography in children between 5 and 15 years of age, with results that varied between 3.16° and 7.6° (22). OCT is a novel method to evaluate ocular torsion, however, its application in children remains unknown. In our study, we enrolled 2-to-6-year-old children and evaluated the ocular torsion angle by fundus photography and GMPE-based OCT. The results were almost similar. There were some children who could not complete the OCT examination due to their non-cooperation and most of the children completed it within 5 min under the supervision of practiced technicians. OCT has the potential to become a new way to quantitatively assess ocular torsion angle and may assist in clinical treatment.

There are many advantages to using OCT for measuring ocular torsion angle. OCT is fast and can be conducted quickly, which could reduce the time patients need to spend in the examination. Furthermore, OCT is relatively easy to use with a simple procedure for both pediatric patients and technicians. It can detect real-time fundus images with high sensitivity and provide precise and specific ocular torsion angles directly. In the future, artificial intelligence technology could be applied in the process to quantitatively assess ocular torsion angle.

Our study has several limitations. The study is a retrospective analysis and we only enrolled 100 patients in our study and further studies are still needed with larger sample sizes. The application of OCT for measuring ocular torsion angle still has some limitations when there are abnormalities in the fundus. The device might identify the wrong location of the fundus and optic disc. Further studies are still needed for these patients.

In conclusion, we quantitatively evaluated ocular torsion angle via fundus photography, combined with Photoshop software, and via OCT. The results showed no significant difference between the two methods. OCT has efficacy in distinguishing ocular extorsion and has broad application prospects in clinical practice in the future.

## Data Availability

The raw data supporting the conclusions of this article will be made available by the authors, without undue reservation.
