# Supplementary figures and images for: The consistency and efficacy of optical coherence tomography for the evaluation of ocular torsion angle in children
Source: Front Pediatr. 2025 Jan 30;13:1519017. doi: 10.3389/fped.2025.1519017 (PMC11821915; doi:10.3389/fped.2025.1519017)

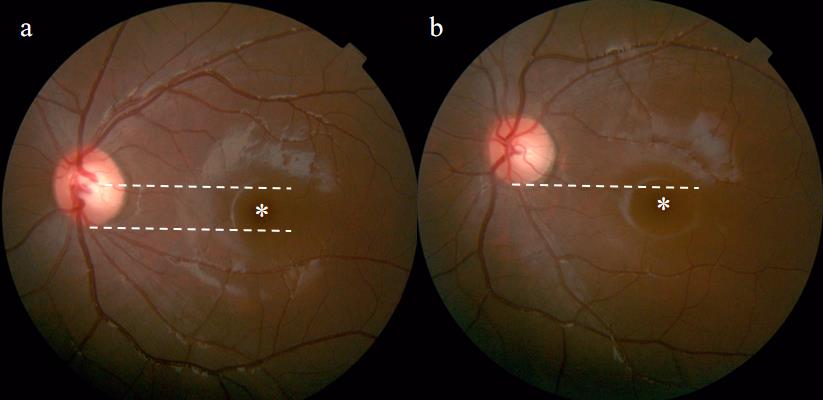

Supplement: Supplementary file 1 [file Image1.jpeg]
